# Long-Term Outcomes After Bariatric Surgery: a Systematic Review and Meta-analysis of Weight Loss at 10 or More Years for All Bariatric Procedures and a Single-Centre Review of 20-Year Outcomes After Adjustable Gastric Banding

**DOI:** 10.1007/s11695-018-3525-0

**Published:** 2018-10-06

**Authors:** Paul E. O’Brien, Annemarie Hindle, Leah Brennan, Stewart Skinner, Paul Burton, Andrew Smith, Gary Crosthwaite, Wendy Brown

**Affiliations:** 1 Centre for Obesity Research and Education, The Alfred Centre, Monash University Clinical School, 99 Commercial Road, Melbourne, 3004 Australia; 2 Centre for Bariatric Surgery, Melbourne, Australia; 3 School of Behavioural and Health Sciences, Centre for Eating, Weight and Body Image, Australian Catholic University, Melbourne, Australia

**Keywords:** Weight loss, Bariatric surgery, Long term, Meta-analysis, 20-year follow-up, Reoperation rates

## Abstract

**Introduction:**

Durability is a key requirement for the broad acceptance of bariatric surgery. We report on durability at and beyond 10 years with a systematic review and meta-analysis of all reports providing data at 10 or more years and a single-centre study of laparoscopic adjustable gastric banding (LAGB) with 20 years of follow-up.

**Methods:**

Systematic review with meta-analysis was performed on all eligble reports containing 10 or more years of follow-up data on weight loss after bariatric surgery. In addition, a prospective cohort study of LAGB patients measuring weight loss and reoperation at up to 20 years is presented.

**Results:**

Systematic review identified 57 datasets of which 33 were eligible for meta-analysis. Weighted means of the percentage of excess weight loss (%EWL) were calculated for all papers included in the systematic review. Eighteen reports of gastric bypass showed a weighted mean of 56.7%EWL, 17 reports of LAGB showed 45.9%EWL, 9 reports of biliopancreatic bypass +/− duodenal switch showed 74.1%EWL and 2 reports of sleeve gastrectomy showed 58.3%EWL. Meta-analyses of eligible studies demonstrated comparable results. Reoperations were common in all groups. At a single centre, 8378 LAGB patients were followed for up to 20 years with an overall follow-up rate of 54%. No surgical deaths occurred. Weight loss at 20 years (*N* = 35) was 30.1 kg, 48.9%EWL and 22.2% total weight loss (%TWL). Reoperation rate was initially high but reduced markedly with improved band and surgical and aftercare techniques.

**Conclusion:**

All current procedures are associated with substantial and durable weight loss. More long-term data are needed for one-anastomosis gastric bypass and sleeve gastrectomy. Reoperation is likely to remain common across all procedures.

## Introduction

The durability of weight loss is the key difference between medical weight loss programs and bariatric surgery [1, 2]. Many medical weight loss programs have reported substantial weight loss but almost none have reported durability beyond 2 years. The Look AHEAD study [3] has been an exception where, with major effort and high costs, a modest effect of 6% total weight loss was reported at a median follow-up of 9.6 years.

Most publications of outcomes after bariatric surgery cover the short term (1–3 years). Some cover the medium term (3–10 years) and just a few provide data on long-term weight loss (10 or more years). Effectiveness and durability are considered key attributes of bariatric surgery when compared with the non-surgical approaches to achieving weight loss. For bariatric surgery to claim a key role in obesity care, strong proof of effectiveness in the long term is needed.

In 2013, we published a systematic review of weight loss at 10 or more years after bariatric surgery, in particular, Roux-en-Y gastric bypass (RYGB), biliopancreatic diversion with or without duodenal switch (BPD/DS) and laparoscopic adjustable gastric banding (LAGB), and we included 15-year outcome data from our patient group after LAGB [4]. That report included 23 datasets from 20 reports available in November 2011. Since that time, the number of published reports providing long-term follow-up data has more than doubled, with 57 datasets now available including some long-term data on sleeve gastrectomy. In addition, we provide very long-term follow-up (20-year) data on our experience with LAGB. To date, only two reports of very long-term data after bariatric surgery are available [5, 6]. We report the systematic review and our own data on 1 January 2018.

## Materials and Methods

### Systematic Review of the Literature

The present report is a synthesis of two systematic reviews. The first search was of the medical literature up to November 2011 and has been published [4]. For the present report, the literature search was extended to December 31, 2017. The pooled data are reported according to the latest Preferred Reporting Items for Systematic Reviews and Meta-Analyses (PRISMA) statement [7].

#### Search Strategy

Articles were identified using the Cochrane Database, Embase, Medline Complete, PubMed, and Scopus electronic databases with the last electronic search conducted on December 31, 2017. Only published full reports were included. Search terms were developed across concepts of “bariatric surgery” ‘AND’ “longitudinal/long term/10 year”. In addition, reference lists of included articles were examined, as were three relevant systematic reviews [8–10]. Hand searching of Obesity Surgery and Surgery for Obesity and Related Disorders was also performed.

#### Eligibility Criteria

Original peer-reviewed English language papers were considered for inclusion. The review considered all types of bariatric surgical procedures except for short-term temporary procedures or experimental procedures (e.g. intragastric balloon, endoscopic duodeno-jejunal bypass sleeve, intragastric stimulation). For inclusion, papers must have reported the weight loss data of at least 10 patients at ≥ 10 years after the initial surgery and expressed the weight outcome as percentage of excess weight loss (%EWL) or percentage of BMI lost (%BMIL) or had provided sufficient information to allow these values to be calculated. Although there has been a recent drive to express weight loss as % total weight loss, almost no data were available in this format. For eligible papers, the data at the final time point that met the inclusion criteria pertaining to participant numbers and weight outcome measurement are reported. We also sought to extract from eligible papers data on operation type, number of patients originally treated and the number at longest eligible follow-up, percentage patients lost to follow up, reoperation rates and weight loss outcome at maximum follow-up. Not all these data are available within the selected papers.

**Fig. 1:**
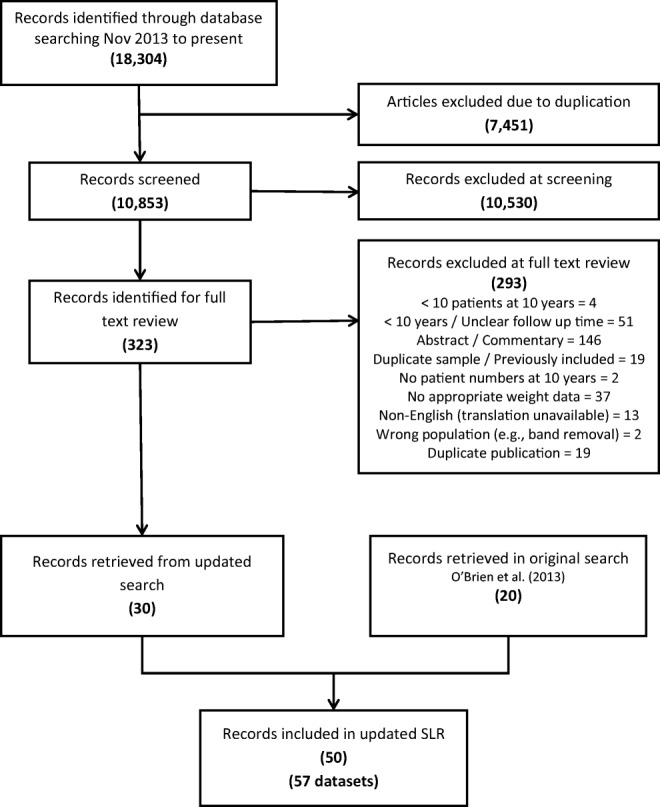
The flow diagram of the systematic review. The eligible articles from the earlier systematic review [4] are added at the level of included studies

One reviewer conducted title and abstract screening with 10% cross-checked by a second reviewer. Both reviewers examined articles identified for full-text review and disagreements concerning inclusion were resolved by joint review. Figure 1 displays the PRISMA search process for the current review with the original review studies added.

### Longitudinal Cohort Study

We have conducted a prospective longitudinal cohort study of all patients having LAGB as a primary bariatric procedure through the Centre for Bariatric Surgery (CBS) in Melbourne from 1994 to the present. The LAP-BAND™ (Apollo Endosurgery, Austin, TX, USA) was used in all cases. The methods of the study are detailed in the earlier report [4]. Briefly, patients treated by LAGB were entered into an electronic medical record for bariatric surgery (LapBase; LapBase Pty Ltd., Melbourne, Australia) at the initial visit. At each visit, the weight was entered. The program provided calculated values for various weight-related parameters (weight loss in kg, BMI change, % total weight loss (% TWL), % excess weight lost (% EWL)) longitudinally and as group reports. Follow-up compliance and group reports for attendance were also provided. Lost to follow up was defined as an absence from the clinic for more than 24 months. In contrast to the 15-year study [4], we did not actively seek contact with those who had not attended to achieve more complete follow-up.

Other clinical data such as reoperation/revision procedures, clinical consultation details, operation reports, endoscopy procedures, barium meals, letters and other reports were stored in LapBase as text or individual items but were not available as group reports. Thus, the details regarding reoperations are the results of hand searching of operation files and have been done for the patients of one surgeon (POB) only.

The outcome measures that are the focus of this report include the weight loss expressed as kg lost, change in BMI, % TWL and %EWL, and reoperation/revisional surgery details. No self-reported weights are included. %EWL was defined as the loss of excess weight in kg above a BMI of 25 kg/m^2^ expressed as a percentage of initial weight.

### Data Analysis

For the systematic review, data for all studies identified in the original and updated searches are presented. The long-term weight loss data for individual studies are summarised in the tables. In addition, weighted means were calculated to examine the long-term %EWL across each surgery technique. As %EWL was calculated from a base of BMI of 25, reports using %EBMIL were treated as equivalent to %EWL.

For studies that reported data eligible for meta-analysis, random-effects analysis was used to calculated pooled mean effect sizes with 95% confidence intervals for each operation type; LAGB (*k* = 13), bypass (*k* = 9), biliopancreatic diversion (*k* = 7) and gastroplasty (*k* = 4). Eighteen studies were excluded from the meta-analysis as they did not report a measure of variance and 2 were single reports of a procedure only. The presence of heterogeneity among effect sizes was assessed using the *Q* statistic and magnitude of heterogeneity with *I*^*2*^. Publication bias was assessed using Egger’s regression symmetry. Comprehensive meta-analysis software (version 3) was used for all analyses.

In the 20-year longitudinal study patient data for weight loss (kg), change in BMI and %EWL were summarised using descriptive statistics and expressed as means ± 95% confidence intervals (CIs). Percentage total weight loss (% TWL) is also presented.

## Results

### Systematic Review

The flow diagram for the search is shown in Fig. 1. The current search covering the period November 2011 to December 2017 yielded 18,304 references. After duplicates were removed, 10,853 references were screened on title and abstract, and 323 were reviewed at full text. Additional data giving separation between the fixed gastric band and the adjustable gastric band from the Swedish Obese Subjects study [11] was provided by personal communication with Dr. Lena Carlsson. Thus, 30 reports containing 34 datasets have been added to the original review which contained 20 reports and 23 datasets to provide a total of 52 reports containing 57 separate datasets for the period 1993 to December 2017. There were two randomised controlled trials [12, 13].

All studies included in the systematic review are listed in Tables 1, 2, 3 and 4 and are summarised in Table 5. There were 18 reports for gastric bypass (Table 1), 16 of which were for the Roux-en-Y (RYGB) and two were for the one anastomosis (OAGB) variant. All gastric bypass combined showed a weighted mean % EWL of 56.7% at 10 or more years with a mean of 55.4% EWL for RYGB and 80.9% EWL for OAGB. The mean EWL for the 17 reports of LAGB was 45.9% (Table 2). For the two RCTs using LAGB, the mean weight loss was 55.9% EWL. There were 11 reports of BPD ± DS which showed a mean of 74.1%EWL (table 3). For the studies of BPD (*N* = 4), the weighted mean was 71.5% EWL whereas for DS (*N* = 7), it was 75.2% EWL. Two reports of sleeve gastrectomy with a total of 79 patients (Table 4) show a mean of 57.0% EWL.

**Table 1:** Gastric bypass

Reference	Type	Initial #	FU %	Duration of FU	# pts at max. years	% EWL at max. years	% reoperation
Fobi, 1993 [14]	RYGB	100	NR	10	46	55	12
Wolfel, 1994 [15]	RYGB	143	71	10	83	49	NR
Pories, 1995 [16]	RYGB	608	97	14	10	49	38
Sugerman, 2003 [17]	RYGB	1025	37	10–12	135	52	NR
Gunther, 2006 [5]	RYGB	195	69	25	72	27	8
Christou, 2006 [18]	RYGB	274	84	12	161	68	NR
Sjostrum, 2007 [19]	RYGB	265	NR	15	10	66	17
Higa, 2011 [20]	RYGB	242	29	10	65	57	32
Angrisani, 2013 [13]	RYGB	24	84	10	21	69	29
Obeid, 2016 [21]	RYGB	328	46	10	134	59	64
Chen, 2016 [22]	RYGB	173	NR	11	78	67	NR
Maciejewski, 2016 [23]	RYGB	1787	82	10	564	56	NR
Monaco-Ferreira, 2017 [24]	RYGB	166	26	10	44	52	NR
Valezi, 2013 [25]	RYGB	211	55	10	116	65	NR
Mehaffey, 2016 [26]	RYGB	1087	61	10	651	52	NR
Kothari, 2017 [27]	RYGB	1402	70	10	191	56	NR
Carbajo, 2017 [28]	SAGB	1200	72	12	29	70	2
Sheikh, 2017 [29]	SAGB*	156	89	11	102	84	14

* indicates silastic ring used

**Table 2:** LAGB

Reference	Type	Initial no.	Follow-up %	Duration of FU	# pts at max. years	%EWL at max. years	% re-operations
Miller, 2007 [30]	Band	554	92	10	154	62	8
Favretti, 2007 [31]	Band	1791	91	11	28	38	19
Naef, 2010 [32]	Band	161	94	10	28	49	20
Himpens, 2011 [33]	Band	154	54	12	36	48	60
Stroh, 2011 [34]	Band	200	84	12	15	33	26
O’Brien, 2013a [4]	Band	3227	81	15	54	47	43
O’Brien, 2013b [12]	Band	40	78	10	31	63	54
Aarts, 2014 [35]	Band	201	99	14	88	38	67
Angrisani, 2013 [13]	Band	27	81	10	22	46	41
Arapis, 2017 [36]	Band	897	90	15	348	42	56
Victorzon, 2013 [37]	Band	60	88	15	16	47	48
Kowalewski, 2017 [38]	Band	107	90	11	37	27	54
Caradina, 2017 [39]	Band	301	79	15	11	38	60
Toolabi, 2015 [40]	Band	80	23	13	18	47	78
Trujillo, 2016 [41]	Band	100	73	12	33	66	55
Vinzens, 2017 [42]	Band	405	85	16	10	50	71
Sjostrom, 2007 [19]	Band	180	NR	15	73	42	52

**Table 3:** BPD and/or DS

Reference	Type	Initial #	Follow-up %	Duration of FU	# pts at max. years	%EWL at max. years	Reoperation %
Hess, 2005 [43]	DS	1300	92	10	167	75	3.4
Scopinaro, 2005 [44]	BPD	312	78	10	243	73	NR
Larrad-Jiminez, 2007 [45]	BPD	343	68	10	65	70	NR
Ballesteros-Pomar 2016 [46]	BPD	299	81	10	34	64	NR
Bolckmans, 2016 [47]	DS	153	79	10	113	94	43
Camerini, 2016 [48]	BPD	120	65	15	79	67	NR
Sethi, 2016 [49]	DS	100	72	10	56	68	37
Pata, 2013 [50]	DS	874	38	10–15 years	328	78	30+
Topart, 2017 [51]	DS	80	73	Jan-00	64	73	11
Marceau, 2007 [52]	DS	1323	NR	Jan-00	284	69	21+
White, 2017 [53]	DS	170	NR	10+	23	61	NR

**Table 4:** Various procedures

Reference	Procedure type	Initial #	Follow-up %	Duration of FU	# pts at max. years	%EWL at max. years	Reoperation %
Arman, 2016 [54]	Sleeve	110	59	11	47	62	32
Felslenreich, 2016 [55]	Sleeve	53	60	10	32	53	36
Fobi, 1993 [14]	Gastroplasty	100	NR	10	43	44	12
Gunther, 2006 [5]	Gastroplasty	33	79	20	18	− 10	NR
Sjostrom, 2007 [19]	Gastroplasty	1369	NR	15	108	44	21
Miller, 2007 [30]	Gastroplasty	563	92	10	154	59	40
Scozzari, 2010 [56]	Gastroplasty	266	70	10	150	60	10
Yu-Hung Lin, 2016 [57]	Gastroplasty	652	NR	10	102	42	13
Canetti, 2016 [58]	Gastroplasty	51	71	10	36	50	NR
Sjostrum, 2007 [19]	Fixed band	196	NR	15	108	32	31
Talebpour, 2012 [59]	Plication	800	NR	10	35	42	NR

**Table 5:** Summary of systematic review of weight loss and reoperation rates

Procedure	No. of reports	Weighted mean % EWL	Mean % EWL range	Reoperation rate range
RYGB	16	55.4	27–69	8–64%
OAGB	2	80.9	70–84	2–14%
LAGB	17	45.9	27–66	8–78%
BPD	4	71.5	64–73	NR
DS	7	75.2	61–94	3–37%
Sleeve	2	57.0	53–62	32–36%
Gastroplasty	7	50.9	− 10–62	10–40%

The single reports of fixed band and plication from Table 6 are not included

*RYGB* Roux-en-Y gastric bypass, *OAGB* one anastomosis gastric bypass, *LAGB* laparoscopic adjustable gastric band, *BPD* biliopancreatic diversion, *DS* duodenal switch,, , NR = not recorded

**Fig. 2:**
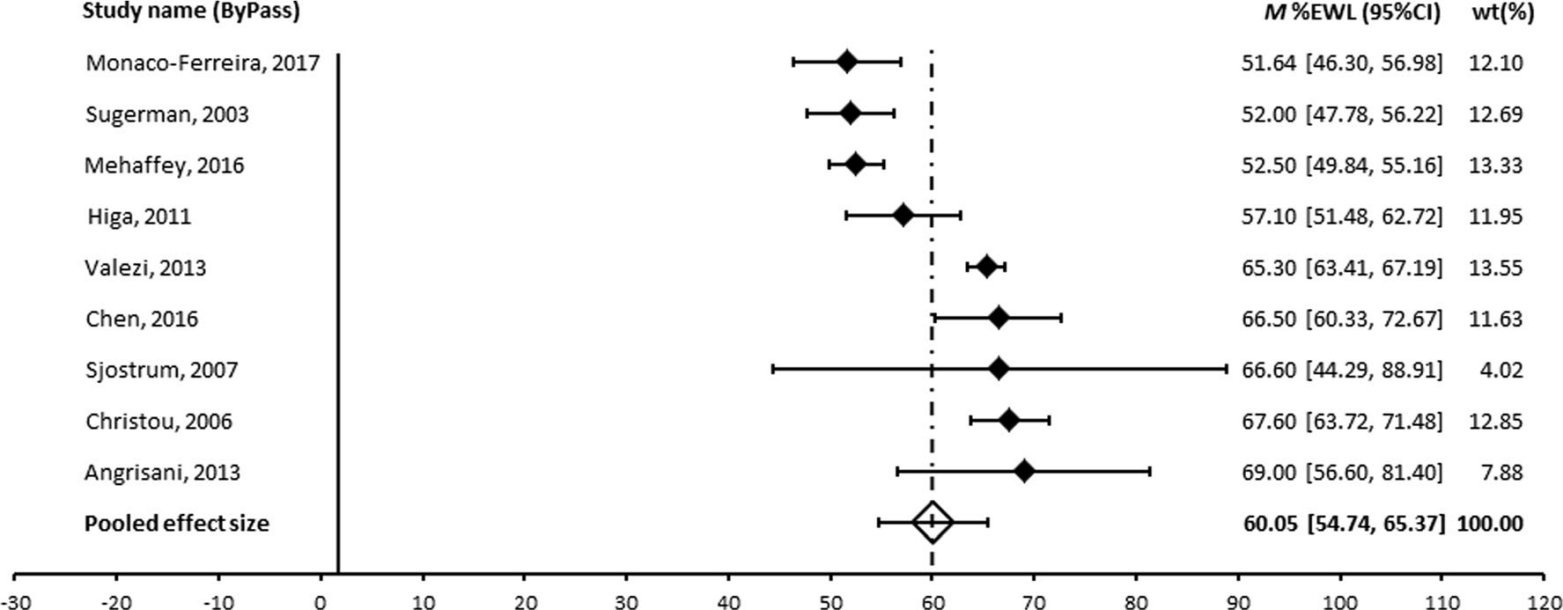
Forest plot of long-term effect of RYGB on %EWL

**Fig. 3:**
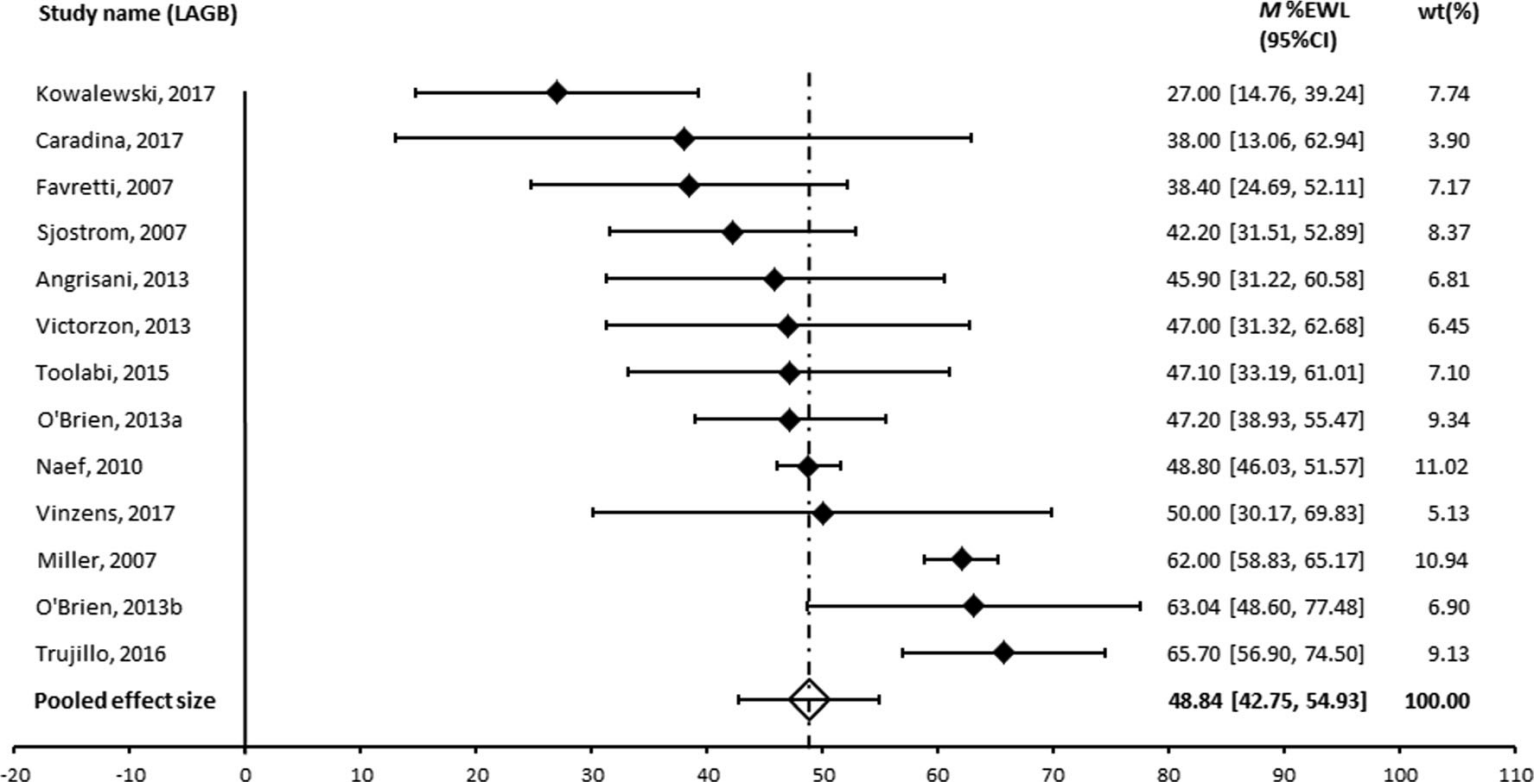
Forest plot of long-term effect of LAGB on %EWL. The RCTs were Angrisani 2013 and O’Brien 2013b

**Fig. 4:**
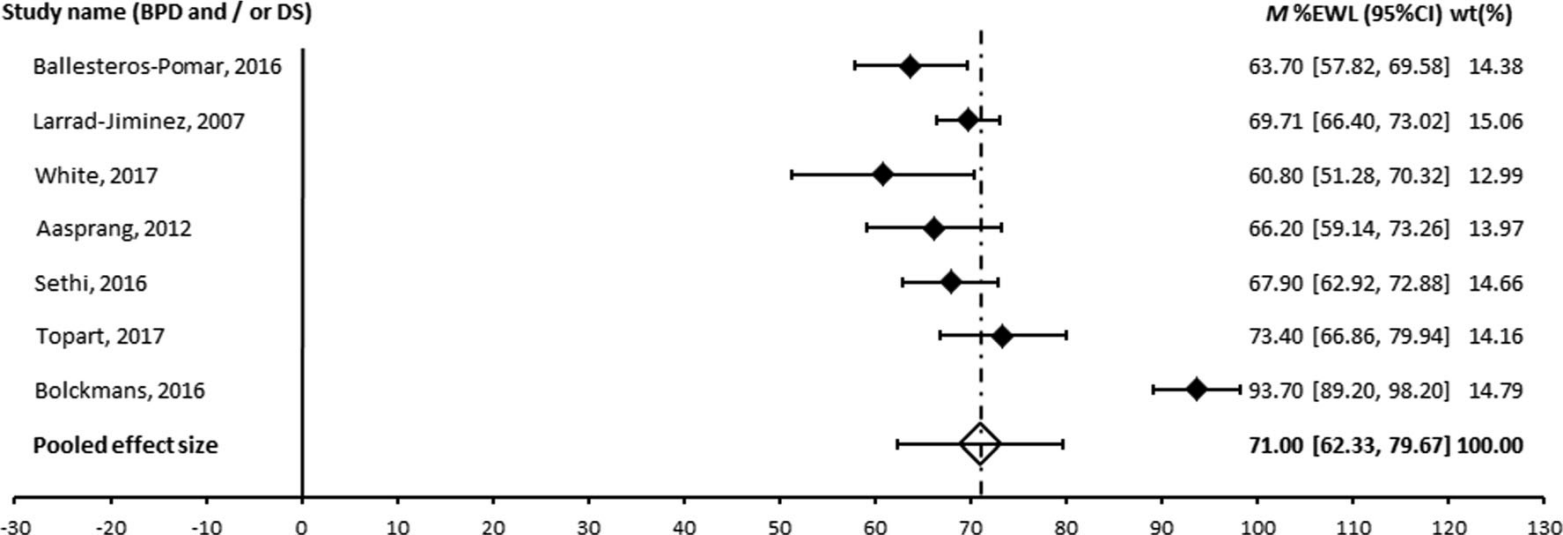
Forest plot of BPD ± DS. The studies of Ballesteos-Pomar 2016 and Larrad-Jiminez 2007 were of BPD alone

A meta-analysis was performed for those procedures where an appropriate measure of variance was provided and where more than two studies were available. The forest plots are shown in Figs. 2, 3 and 4. The pooled effect size was 60% for RYGB, 49% for LAGB and 71% for BPD ± DS. Egger’s regression was non-significant for all operation types suggesting the results are not affected by publication bias. Heterogeneity was large across all operation types [60]. Patient numbers at follow-up, assessment of heterogeneity and publication bias are shown in Table 6.

**Table 6:** Meta-analysis pooled effect sized (%EWL), heterogeneity and publication bias

Operation	Studies (N)	Patients at follow-up	Pooled effect size (95% CI)	*Q*	*I* ^2 (%)^	Egger’s regression intercept
RYGB	9	1326	60.05 (54.74, 65.37)	105.63*	92.43	− 3.33, *p* = .575
LAGB	13	485	48.84 (42.75, 54.93)	80.79*	85.15	− 1.31, *p* = .262
BPD/DS	7	393	71.00 (62.33, 79.67)	108.63*	94.48	− 3.33, *p* = .575
Gastroplasty	4	448	53.54 (45.78, 61.30)	39.01*	92.31	− 5.13, *p* = .492

*p < .001

Pooled effect sizes were compared across surgery types. BPD/DS produced significantly greater weight loss compared to all other operation types: RYGB (*Q* (1)=4.45, *p* < .05), gastroplasty (*Q* (1)=8.66, *p* < .01), LAGB (*Q* (1)=17.06, *p* < .001) and sleeve (*Q* (1)=4.46, *p* < .05). RYGB produced significantly greater weight loss compared to LAGB (*Q* (1)=7.62, *p* < .01).

There were no differences in %EWL between RYGB and sleeve (*Q* (1)=.30, *p* = .585), sleeve and gastroplasty (*Q* (1)=0.29, *p* = .590), or between LAGB and sleeve (*Q* (1)=2.01, *p* = .156), or LAGB and gastroplasty (*Q* (1)=0.95, *p* = .330).

### CBS Longitudinal Cohort Study

A total of 8378 patients (77.4% female) were treated by primary LAGB procedure. They had a mean age of 42 years (range 14–77 years) and had a mean initial weight of 121.2 kg and a mean initial BMI of 43.2 kg/m^2^.

Follow-up has been maintained for 54% of patients overall. The loss to follow-up was higher as the follow-up period lengthened. The percent follow-up of each annual cohort is shown in Table 6 and 7.

**Table 7:** Single-centre review of weight loss with up to 20 years of follow-up after LAGB

Year	No. of patients	Weight loss (kg)	95% CI	% total weight loss	Change of BMI (units)	95% CI	%EWL	95% CI	% follow-up
0	8378	0		0	0		0		
1	7817	21.9	0.28	18.1	7.8	0.1	45.8	0.53	92.5
2	7264	24.6	0.34	20.4	8.8	0.12	52.6	0.62	89.1
3	6877	24.5	0.39	20.3	8.7	0.14	51.4	0.66	85.0
4	6006	24.0	0.43	19.9	8.6	0.15	49.3	0.73	69.4
5	5235	23.7	0.47	19.5	8.5	0.17	47.7	0.84	68.6
6	4570	23.2	0.50	19.2	8.3	0.18	46.4	0.93	55.0
7	3917	22.9	0.56	19.0	8.2	0.20	45.6	0.97	46.6
8	3333	23.1	0.60	19.1	8.3	0.21	45.5	1.1	43.9
9	2768	22.9	0.65	19.0	8.2	0.23	44.8	1.1	41.9
10	2275	23.2	0.73	19.4	8.3	0.25	45.5	1.3	39.0
11	1860	23.6	0.81	19.5	8.5	0.29	45.6	1.4	43.8
12	1472	24.1	0.91	20.1	8.7	0.33	46.7	1.6	36.6
13	1147	24.4	1.03	20.4	8.8	0.36	46.8	1.8	41.3
14	827	25.4	1.26	20.9	9.1	0.44	47.7	2.0	33.8
15	599	25.4	1.47	21.2	9.1	0.5	47.9	2.4	34.0
16	436	25	1.75	21.1	9.0	0.63	47.1	3.0	36.5
17	292	26.4	2.3	22.0	9.5	0.9	48.3	3.8	27.9
18	181	27.2	3.0	22.2	9.6	1.1	47.3	4.4	23.8
19	95	27.2	4.0	22.1	9.5	1.4	46.1	6.0	24.6
20	35	30.1	9.2	22.2	10.6	3.2	48.9	13.9	25.0

### Mortality

There have been no deaths associated with any primary bariatric procedure or any subsequent revisional procedure. To our awareness, there have been no late deaths that should be attributed to the procedure.

### Weight Loss

**Fig. 5:**
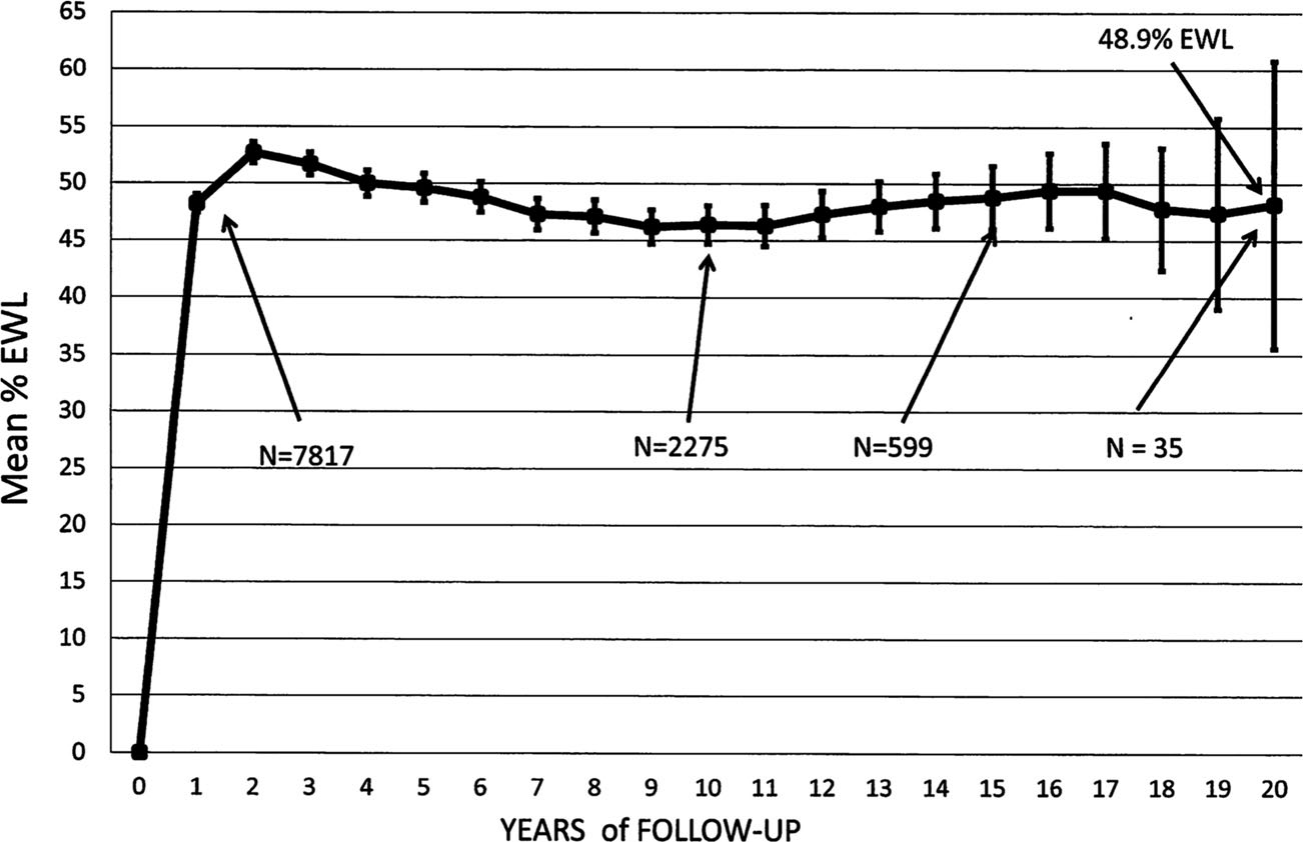
The weight loss expressed as %EWL ± 95% CI for the 20-year period of follow-up after LAGB. The initial *N* = 8378 patients. At 20 years, there was 30.1 kg weight loss and 22.2%TWL

The weight loss over time is shown as kg weight loss, change in BMI, % TWL and %EWL in Table 7 and as %EWL in Fig. 5. Table 7 also shows the number of patients at each annual time point. The weight loss had reached a peak at 2-year follow-up and remained relatively stable from 2 to 20 years with mean weight loss for this period of 24.8 kg representing 47.2 %EWL. Thirty-five patients have completed 20-year follow-up and have maintained a mean loss of 30.1 kg (48.9% EWL, 22.2% TWL) at that time. Nineteen of the 35 patients (54%) had retained a loss of more than 50% of their excess weight at 20 years. Although the number of patients completing 20-year follow-up is modest, much larger numbers have completed 15–19 years as shown on Table 7 and show a very similar weight loss status (Fig. 5).

### Revisional Surgery

The revisional surgery rates for the 3554 patients treated by one surgeon (POB) are shown in Table 8. Enlargements of stomach above the band remain the most common indication for revision and include posterior slips, anterior slips and symmetrical enlargements. Whereas in our initial experience posterior slip was common, the shift to the pars flaccida pathway for band placement has almost totally removed that problem [61]. Anterior slip has been reduced by improved anterior fixation of the stomach across the band and symmetrical enlargement remains the main anatomical abnormality leading to revision. Of the 214 revisions after initial placement of a Lap-Band AP version, introduced in 2006, 197 procedures were for symmetrical enlargement (92%), 12 procedures for anterior slip (5.6%), and 5 procedures for posterior slip (2.3%). The need for revisional surgery for proximal enlargements has decreased markedly in the last 11 years, in association with the use of the Lap-Band AP and improved patient education, moving from more than 50% during the early era to 11.3% for the last 12-year period. Erosion of the band into the gastric lumen has occurred in 114 patients with an overall rate of 3.2%. A detailed clinical report on the first 100 erosions has been published [62]. There has been a marked reduction in this problem in association with the change of band design, decreasing from 6% during the Lap-Band 10 cm era to less than 0.7% in the Lap-Band AP era (Table 8).

**Table 8:** Reoperations/revisions during the follow-up period

	Total period	Lap-Band 10 cm era	Lap-Band AP era
	1994–2017 (*N* = 3554)	1994–2005 (*N* = 1658)	2006–2017 (*N* = 1896)
Enlargements	1063 (29.9%)	847 (51.7%)	214 (11.3%)
Erosions	114 (3.2%)	101 (6.1%)	13 (0.69%)
Explantations/conversion	305 (8.6%)	199 (12%)	104 (5.5%)
Port/tubing	760 (21.4%)	599 (35.7%)	161 (8.5%)

Explanations have occurred in 8.6% of patients. The most frequent reason is patient request due to food intolerance.

Port and tubing problems represent a separate and relatively minor array of events. Common problems were needle stick injury to the tubing adjacent to the port, tubing being rubbed through, breakage in the tubing generally adjacent to a metal connector and inaccessibility or rotation of the port.

## Discussion

The meta-analysis of published long-term outcomes shows the principal bariatric surgical procedures provide substantial and durable weight loss. The most impressive outcome came from the BPD or its DS variant with a pooled effect size of 71.0% EWL followed by RYGB with 60% EWL and LAGB with 49% EWL.

Data were insufficient for a meta-analysis of the sleeve gastrectomy but it generated a weighted mean of 57% EWL from the two small studies that were included in the systematic review. Similarly, OAGB had just two studies which showed a high weighted mean of 81% EWL. It is appropriate to be cautious when just two studies are available and therefore, we wait expectantly for additional long-term data on both sleeve and OAGB.

The results shown would appear to confirm the greater effectiveness and durability of bariatric surgery compared to optimal medical therapy. The Look AHEAD study which arguably provides the best example of what can be achieved by a concentrated and continuing process of an intensive lifestyle intervention, achieved approximately 15% EWL at 8 years [3].

Whilst it is reassuring to note long-term effectiveness, the quality of most of the studies was low with a general lack of control data, numerous data gaps including percentage follow-up, reoperation rates, perioperative mortality and morbidity and even the measures of variance were absent in several reports. There is a critical need for higher levels of evidence. Only two RCTs have been included [12, 13]. Angrisani et al. compared RYGB and LAGB and showed 69% EWL and 46% EWL respectively [13]. In the Australian study which compared LAGB to optimal medical therapy, the LAGB group showed 63% EWL at 10 years. More recently, three important RCTs of 5-year outcomes with RYGB versus sleeve gastrectomy have been published [63–65]. The SLEEVEPASS study [63] reported 57% EWL for RYGB and 49% EWL for sleeve at 5 years. The SM_BOSS study [64] reported 68% excess BMI lost (EBMIL) for RYGB and 61% EBMIL for sleeve. The STAMPEDE study [65] reported 21.7% TWL for RYGB and 18.5% TWL for sleeve. We look forward to longer follow-up from these and other such studies. It is likely that the broad acceptance of bariatric surgery will not occur until these additional higher quality data become available.

The CBS longitudinal cohort study shows substantial weight loss to be present by 2 years and this effect remained relatively constant for the subsequent 18 years, finishing with a final value of 48.9% EWL and 22.2% TWL at 20 years (*N* = 35). Only one other study has reported longer follow-up. Gunther et al. [5] followed 198 patients for up to 25 years. For transected RYGB patients, they reported 29.9% EWL at 20 years (*N* = 53) and 25.5% EWL at 25 years (*N* = 62) [5]. They reported a net weight gain at 25 years for their gastroplasty patients. The adjustability of the LAGB could be a key factor in maintaining stable weight loss status compared to the progressive fading of effect with non-adjustable stapling procedures.

Revisional surgery was not always reported in the studies within the systematic review but, when provided, showed reoperation was common after all surgical options. The need for reoperation has been seen to be a weakness of LAGB and the reoperation rate for the longitudinal cohort study was high during the Lap-Band 10 cm era with over 50% needing revision for proximal gastric enlargements above the band. The need dropped sharply during the Lap-Band AP era to just over 10%. Part of this lower incidence would reflect lead-time bias. Other factors include an improved band design and better understanding of how the band works [66–68] leading to more appropriate patient education and aftercare support.

In the review of our 15-year outcomes [4], we had provided a table identifying important modifications of the band, its placement and aftercare up to 2012 that were leading to improved outcomes. Two recent additions to that list are worth noting. The first is the use of the mesh fixation of the access port. This technique has added several advantages of less post-operative pain, easier adjustments of the band in the office and less reoperation for needle stick injury or unstable port position. As a consequence, attendance at aftercare is better, leading to improved outcomes. The second important change has been a better focus on slow eating with the strict adherence to a minimum of 1-min duration between bites [68]. This has enabled better control of appetite and reduced incidence of symmetrical enlargement.

The systematic review has shown that all procedures have a substantial need for re-operative surgery and the levels of reoperation for LAGB during the Lap-Band AP at CBS are within the range of other bariatric procedures in the systematic review.

Maintaining completeness of follow-up will always be a major challenge for long-term follow-up studies. Direct contact follow-up was available in the CBS study of 58% overall. This is less than 81% follow up achieved in the 15-year follow-up report [4], in which study we made dedicated effort to bring in as many additional patients as possible. No specific efforts were made for the present study. The 58% achieved for the full 20-year cohort can be compared to the 57% direct contact follow-up achieved at 7 years for the LABS-2 study [69, 70], a well-funded and strongly committed clinical trial.

The studies have several limitations. The quality of the data for the systematic review was low with only two randomised controlled trial available. A total of 18 reports accepted for the systematic review had to be excluded from the meta-analysis because they provided no appropriate measure of variance. The secondary aims of the systematic review could not always be achieved as data on mortality, percentage patients lost to follow up and reoperation rates were not provided in many cases. The relative lack of long-term data for sleeve gastrectomy is of concern as it has already become the most common bariatric procedure [71], yet lacking strong evidence of durability. Notably, the 5-year weight loss from one of the RCTs using the sleeve [63] is the same as our data on the 20-year weight loss for LAGB.

In summary, RYGB, LAGB and BPD/DS lead to substantial weight loss which continues for at least 10 years. Each has an effect size three to four times that of optimal non-surgical therapy. The reoperation rate is significant for each procedure. Long-term data on sleeve gastrectomy and OAGB are modest at this time. Data on LAGB from a single centre shows stability of weight loss at just less than 50% EWL through 20 years. There has been a substantial reduction in reoperation rates associated with improved band design and better quality of patient education due to with improved understanding of how the band works. Guidance to the obese patient should balance durable effectiveness with risks, costs, health benefits and effect on quality of life.
